# Facile Fabrication of Superhydrophobic and Flame-Retardant Coatings on Cotton Fabrics

**DOI:** 10.3390/polym14235314

**Published:** 2022-12-05

**Authors:** Shiwei Li, Luyan Yu, Jianhua Xiong, Ying Xiong, Shuguang Bi, Heng Quan

**Affiliations:** 1 Hubei Key Laboratory of Biomass Fibers and Eco-Dyeing & Finishing, College of Chemistry and Chemical Engineering, Wuhan Textile University, Wuhan 430200, China; 2 Jiangsu Engineering Research Center of Textile Dyeing and Printing for Energy Conservation, Discharge Reduction and Cleaner Production (ERC), Soochow University, Suzhou 215123, China; 3 High-Tech Organic Fibers Key Laboratory of Sichuan Province, Chengdu 610037, China

**Keywords:** superhydrophobicity, flame retardancy, cotton fabric, layer-by-layer assembly

## Abstract

The hydrophilicity and inherent flammability of cotton textiles severely limit their usage. To solve these drawbacks, a superhydrophobic and flame-retardant (SFR) coating made of chitosan (CH), ammonium polyphosphate (APP), and TiO_2_-SiO_2_-HMDS composite was applied to cotton fabric using simple layer-by-layer assembly and dip-coating procedures. First, the fabric was alternately immersed in CH and APP water dispersions, and then immersed in TiO_2_-SiO_2_-HMDS composite to form a CH/APP@TiO_2_-SiO_2_-HMDS coating on the cotton fabric surface. SEM, EDS, and FTIR were used to analyze the surface morphology, element composition, and functional groups of the cotton fabric, respectively. Vertical burning tests, microscale combustion calorimeter tests, and thermogravimetric analyses were used to evaluate the flammability, combustion behavior, thermal degradation characteristics, and flame-retardant mechanism of this system. When compared to the pristine cotton sample, the deposition of CH and APP enhanced the flame retardancy, residual char, heat release rate, and total heat release of the cotton textiles. The superhydrophobic test results showed that the maximal contact angle of SFR cotton fabric was 153.7°, and possessed excellent superhydrophobicity. Meanwhile, the superhydrophobicity is not lost after 10 laundering cycles or 50 friction cycles. In addition, the UPF value of CH/APP@TiO_2_-SiO_2_-HMDS cotton was 825.81, demonstrating excellent UV-shielding properties. Such a durable SFR fabric with a facile fabrication process exhibits potential applications for both oil/water separation and flame retardancy.

## 1. Introduction

Cotton fabric is widely used owing to its unique comfortable and breathable, non-toxic, and biodegradable properties [1,2]. However, the inherent flammability of cotton fabric can extremely restrict its applications. Therefore, it is necessary to improve its flame retardancy [3,4]. A variety of techniques have been used to create flame-resistant cotton fabric, including sol-gel treatment, UV-curable, pad-dry-cure, plasma, layer-by-layer (LBL) assembly, and others [5,6]. These techniques all have the similar goal of stabilizing fire retardancy on the cotton fibers’ surface. The LBL assembly technique, which employs the principle of alternate layers deposited by positive and negative electrolytes, is one of the most effective surface modification methods to fabricate flame-retardant coatings due to its practicality and adaptability [7,8].

Traditional flame retardants contain halogen elements and are gradually being eliminated because of the toxic and corrosive gases that are generated. Flame retardants containing N and P are used to replace those flame retardants containing halogen elements. Thus, many materials have been chosen to equip cotton fabric with flame-retardancy, such as phytic acid, ammonium dihydrogen phosphate, ammonium polyphosphate (APP) and so on. With low toxicity and great thermal stability, APP is an intumescent and halogen-free flame retardant that can encourage the formation of intumescent char layers on substrates. By isolating the air and dampening the fire, this layer provides good flame retardancy to the substrates [9,10,11]. In dilute acid solutions, chitosan (CH) can produce positive charges [12]. APP is a popular inorganic flame retardant that may produce negative charges in aqueous solutions. Furthermore, because CH contains several reactive hydroxyl groups, it may be employed as a carbon source to create an intumescent flame retardant with APP. As a result, CH and APP may be employed as polycation and polyanion in LBL assembly to create flame-resistant cotton textiles [13,14].

However, the durability of flame-retardant fabrics is still a big challenge because flame retardants are generally hydrophilic. The flame-retardant components of the cotton fabric may be lost in water. Hence, enhancing the water repellency of a cotton fabric surface is helpful to maintain long-term flame retardancy. Moreover, multifunctional cotton fabric with both flame-retardant and superhydrophobic properties has attracted considerable attention [15,16].

Superhydrophobic surfaces are defined as having a water contact angle higher than 150° and a water sliding angle lower than 10°. Superhydrophobic coatings have been extensively researched and applied in self-cleaning, oil/water separation, anti-condensation, anti-fogging, and anti-icing [17,18,19,20,21,22]. Inspired by the lotus leaf effect, numerous studies have shown that superhydrophobicity can be obtained by the combination of micro/nano scale surface roughness and a low-surface-energy coating [23,24]. To endow the fabric with superhydrophobic property, it is usually designed hierarchical roughness on a material possessing low surface energy, or modified a rough surface with a low-surface-energy material [25]. Based on this principle, many superhydrophobic surfaces have been successfully fabricated through various approaches, such as electrospinning, sol-gel processing, phase separation, spray coating and self-assembly [26,27,28,29,30,31,32]. Nevertheless, most of these methods require complicated conditions or specialized machines, limiting their practicality. In addition, fluorine-containing compounds are commonly used to reduce surface energy, but these are expensive and pose risks to human health and the environment. The sol–gel method appears to be promising for producing superhydrophobic textiles. Surface roughness is created by carefully controlling the hydrolysis (sol) and condensation (gel) reactions of tetraethoxysilane or tetrabutyl titanate to introduce inorganic nanoparticles such as SiO_2_ and TiO_2_, and then modifying the surface with the low-surface-energy material. Superhydrophobic cotton fabric is obtained by the incorporation of SiO_2_ nanoparticles and subsequent hydrophobization. However, it is a two-step process in which the modification of low surface energy and surface roughness are performed separately. Therefore, this study focuses on creating a one-step, non-fluorinated technique for superhydrophobic cotton fabric finishing [33,34,35,36,37,38].

Nano TiO_2_ has the advantages of low price, non-toxicity and simple preparation, which promotes its wide range of applications. Nano TiO_2_ can endow cotton fabric with excellent UV-shielding performance, which avoids some disadvantages of traditional organic UV-shielding agents, such as short service life, poor stability, and environmental issues. However, TiO_2_ may decompose organic substrates owing to photocatalysis when treated directly on the surface of the fabric, causing damage to the fabric and degrading its application properties. As a result, TiO_2_ nanoparticles are coated with inert materials, allowing them to absorb UV light while also inhibiting photocatalysis, making them more environmentally friendly. In addition, nano SiO_2_ has excellent mechanical characteristics, thermal stability, and so on. Encapsulating nano TiO_2_ with nano SiO_2_ (TiO_2_-SiO_2_) blocks the interaction between the nano TiO_2_ and fabric, lowering photocatalytic activity and protecting the fabric. Furthermore, when TiO_2_-SiO_2_ nanocomposite particles are applied to the fabrics and modified by low-surface-energy substances, the nano SiO_2_ coating can prevent the photocatalytic degradation of low-surface-energy substances by nano TiO_2_, allowing the fabrics to have both UV resistance and superhydrophobic properties. TiO_2_-SiO_2_ may be utilized in a variety of applications, including sun and rain umbrellas, open-air tents, advertising umbrellas, as well as for military, industrial, and medicinal purposes [39].

In this work, a superhydrophobic and flame-retardant CH/APP@TiO_2_-SiO_2_-HMDS (SFR) cotton fabric was fabricated by CH/APP coating via LBL assembly, and subsequent dip-coating with TiO_2_-SiO_2_-HMDS. The LBL assembly method was used because it was easy and convenient to control the thickness of the flame-retardant coating by altering the number of assembly layers, thereby adjusting the flame-retardant characteristic of the fabric. The hydrophobic TiO_2_-SiO_2_-HMDS particles were prepared by the sol–gel method, and then the superhydrophobic finishing of cotton fabric was carried out by the one-step method, which saved process steps. TiO_2_-SiO_2_-HMDS not only endowed fabrics with excellent UV-shielding properties, but also reduced the photocatalytic activity of TiO_2_ and protected the low surface energy materials and fabrics, so that the fabrics possessed UV-shielding and superhydrophobic properties. Furthermore, superhydrophobic finishing could improve the washing resistance of flame-retardant components, expanding the application range of cotton textiles. Fourier transform infrared (FTIR) spectroscopy, X-ray diffraction (XRD), scanning electron microscopy (SEM), and energy-dispersive spectrometry (EDS) were used to analyze the structure and chemical composition of manufactured textiles. Thermogravimetric analysis (TGA), microscale combustion calorimetry (MCC), and a vertical burning test were used to assess thermal stability and flame retardancy. The properties of superhydrophobicity, wettability, self-cleaning, UV-shielding, and stability were also investigated.

## 2. Experimental

### 2.1. Materials

APP was obtained from Shanghai Aladdin Biochemical Technology Co., Ltd. (Shanghai, China). CH (deacetylation degree ≥ 95%) and nano titanium dioxide (100 nm) were obtained from Shanghai Aladdin Biochemical Technology Co., Ltd. (Shanghai, China). Hexamethyldisilamine (HMDS) and polyvinylpyrrolidone (PVP) were obtained from Shanghai Maclin Biochemical Technology Co., Ltd. (Shanghai, China). Tetraethoxysilane (TEOS) was purchased from Sinopharm Chemical Reagent Co. Ltd. (Shanghai, China). Cotton fabrics were purchased from local markets. All the reagents were used as received.

### 2.2. Preparation of SFR (CH/APP@TiO_2_-SiO_2_-HMDS) Cotton Fabric

**Figure 1:**
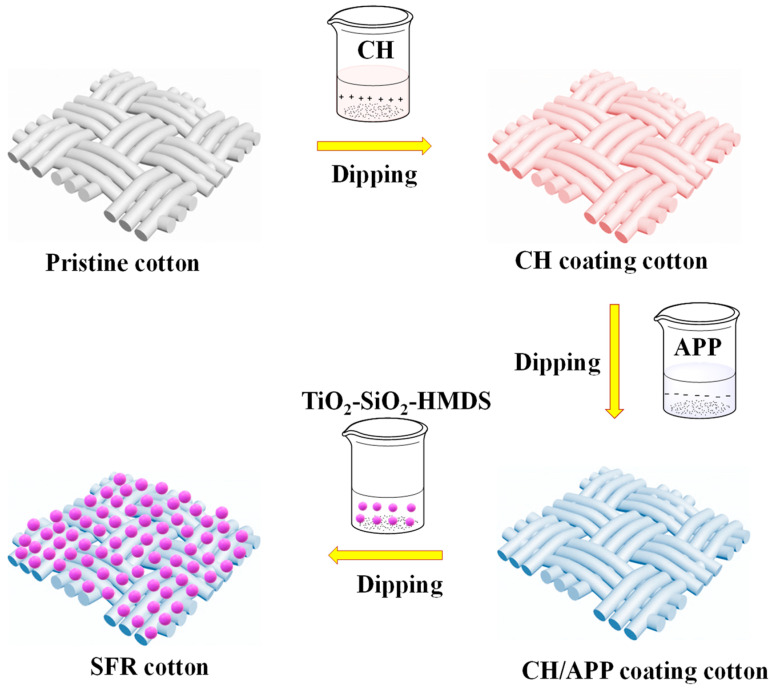
Schematic illustration of the fabrication process of SFR coating on cotton fabric.

Figure 1 depicts a schematic illustration of the fabrication process for an SFR coating on cotton fabric. The pristine cotton fabric was first cleaned before being immersed in 0.75% aqueous solution of CH for 10 min. After being rinsed with deionized water and dried for 1 h in an oven at 80 °C, the fabric was immersed in 1.5% APP aqueous solution for 10 min, and then rinsed with deionized water and dried in the oven at 80 °C for 1 h. The process was repeated until there were five CH/APP deposition layers on the cotton fabric.

To begin with, 0.75 g nano TiO_2_ was added into 50 mL absolute ethanol. Then 0.01 g PVP was added into the absolute ethanol, and dispersed by ultrasound for 20 min. The evenly dispersed nano TiO_2_ was transferred to a three-neck flask; 4 mL tetraethyl orthosilicate (TEOS), 5 mL ammonia and 30 mL absolute ethanol were added into the three-neck flask in 30 min, then reacted at 55 °C for 3.5 h. Subsequently, 7.5 mL HMDS was added, and reacted at 55 °C for 1.5 h to obtain TiO_2_-SiO_2_-HMDS sol solution. The TiO_2_-SiO_2_-HMDS sol solution was aged for 24 h at room temperature. After that, the CH/APP coated cotton fabric was dipped into the TiO_2_-SiO_2_-HMDS sol solution for 15 min, and dried in an oven at 80 °C. SFR cotton fabric was obtained. Except for samples with specially marked dosages, other samples without a marked experimental dosage were prepared under this condition.

### 2.3. Characterizations

FTIR spectra were collected using a spectrometer (Tensor 27, Bruker Optik GmbH, Salbruken, Germany) with a scan range of 4000-400 cm^−1^ and a resolution of 2 cm^−1^.The XRD patterns of samples were tested by wide-angle X-ray diffractometry (XRD, Ultima IV, Rigaku Corporation, Tokyo, Japan), using filtered Cu-Kα radiation (40 kV, 40 mA). The 2θ angle of the diffractometer was stepped from 5° to 80° at a scan rate of 5°/min. The thermogravimetric analysis (TGA) was performed by a thermogravimetric analyzer (TGA, Mettler Toledo International Co., LTD, Zurich, Switzerland) from 30 °C to 800 °C at a heating rate of 10 °C/min under a nitrogen atmosphere with a flow rate of 10 mL/min. The surface morphologies and energy dispersive spectrometer (EDS) were determined by scanning electron microscope (JSM IT500A, JEOL Ltd., Tokyo, Japan) with an acceleration voltage of 10.0 kV. The UV-resistant performance of cotton fabrics was tested by an ultraviolet transmittance spectrometer (UV2000, Labsphere, North Sutton, NH, USA). MCC tests were conducted on a microscale combustion calorimeter (MCC-2, Govmark Organization Inc., New York, NY, USA), according to the ASTMD7309-2007a standard. The samples were heated at a rate of 1 °C/min from 50 °C to 750 °C. The vertical flame test of the textiles was performed using a fabric flame-retardance tester (YG815B, Ningbo Textile Instrument Factory, Ningbo, China) in accordance with the GB/T 5455-2014 standard. At room temperature, static contact angles (CA) of various pristine and coated materials were measured using a contact angle analyzer (5 μL, DSA 20, KRUSS GmbH, Hamburg, Germany). Five measurements were performed for each sample and the average contact angle was obtained. Self-cleaning ability: samples were put in a glass dish and tilted at a 20° angle. After scattering some reactive red dye powders on the surface, water was dumped on it to wash the powders away. Antifouling property: samples were arranged horizontally on a table, and then typical pollutants (such as active red dye, milk, coffee, and orange juice) were poured across the surfaces. Laundering durability of the cotton fabric was tested according to AATCC Test Method 61-2006. In one laundry cycle, the sample was submerged in a 40 °C solution containing 2 g/L of standard reference detergent with a liquor ratio of 50:1 and spun in the machine at 40 r/min for 30 min before being washed with deionized water for 3 min for the next cycle. These three cycles (90 min) are approximately equivalent to 10 times soft washing. Abrasion resistance of the cotton fabric was tested according to ISO105-X12:2001 using the color fastness tester (Y571N, Nantong Hongda Experimental Instrument Co., Ltd., Nantong, China).

## 3. Results and Discussion

### 3.1. Surface Morphology and Chemical Composition

**Figure 2:**
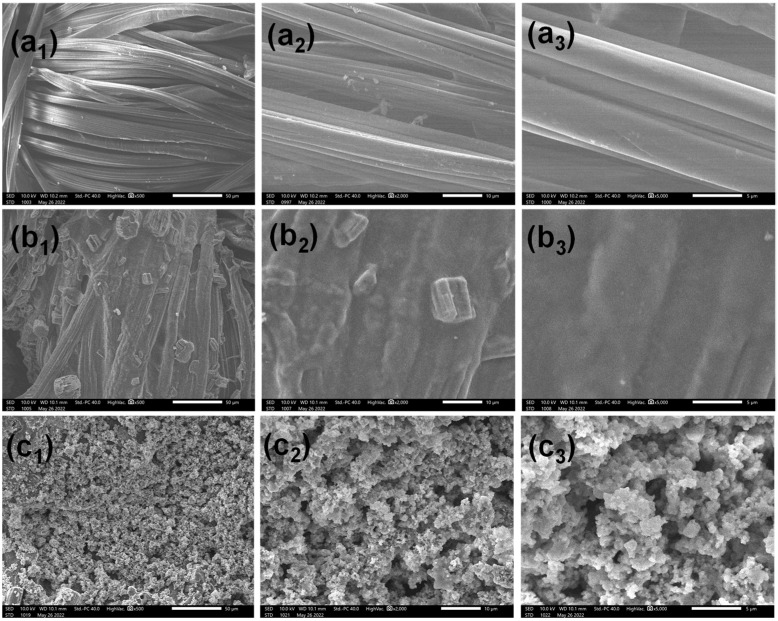
SEM images of pristine cotton (**a_1_**–**a_3_**), CH/APP cotton (**b_1_**–**b_3_**), CH/APP@TiO_2_-SiO_2_-HMDS cotton (**c_1_**–**c_3_**).

The SEM images of pristine cotton, CH/APP cotton, and CH/APP@TiO_2_-SiO_2_-HMDS cotton fabric at three different magnifications are shown in Figure 2. The surface morphology of pristine cotton fabric was generally smooth and plain, as seen in the Figure 2(a_1_–a_3_). In contrast, after progressively assembling CH and APP on cotton fabric, the surface became rough because the fibers were completely and uniformly covered by the assembly, as seen in Figure 2(b_1_–b_3_). Because chitosan is flexible, there are no fractures in the cotton fabric. The cotton fabric surface was coated with a consistent micro/nano scale rough structure coating after being deposited with TiO_2_-SiO_2_@HDMS composite, as illustrated in Figure 2(c_1_–c_3_). The SEM results demonstrated that the surface of the CH/APP@TiO_2_-SiO_2_-HMDS cotton fabric had a totally uniform CH/APP coating and a micro/nano scale rough structure with low-surface-energy substance coating, which were responsible for the flame-retardant and superhydrophobic properties.

EDS analysis was used to investigate the chemical composition of the cotton fabric surfaces, and the results are presented in Table 1. As shown in Table 1, only C and O elements existed on the pristine cotton. Except for C and O elements, the presence of the N and P elements on the CH/APP cotton indicated that CH and APP have been successfully loaded onto the cotton fabric. C, O, Si and Ti elements were detected on the CH/APP@TiO_2_-SiO_2_-HMDS cotton fabric. It showed that the TiO_2_-SiO_2_-HMDS composite was deposited on the surface of cotton fabric. Therefore, the EDS results indicated that CH, APP, and TiO_2_-SiO_2_-HMDS were successfully loaded onto the surface of the cotton fabric.

**Figure 3:**
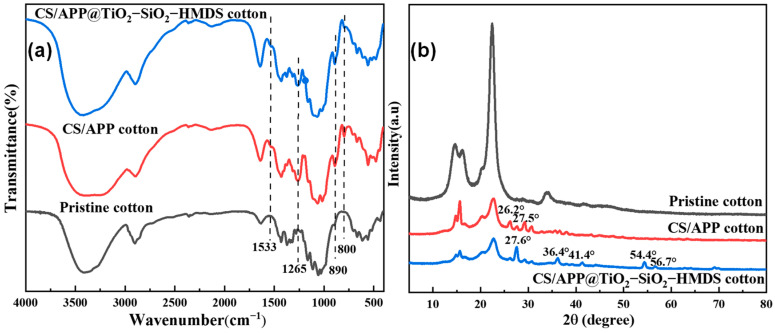
(**a**) FTIR of spectra of the pristine cotton, CH/APP cotton and CH/APP@TiO_2_-SiO_2_-HMDS cotton, (**b**) X-ray diffraction spectra of the pristine cotton, CH/APP cotton and CH/APP@TiO_2_-SiO_2_-HMDS cotton.

The FTIR spectra of the pristine fabric, CH/APP fabric, and CH/APP@TiO_2_-SiO_2_-HMDS fabric was displayed in Figure 3a. Chitosan was positively charged under acidic conditions, and cotton fabric was negatively charged by hydroxyl ionization in an aqueous solution. The cotton fabric was bonded to the chitosan by electrostatic gravitational forces. At the same time, chitosan had un-ionized amino groups that could form hydrogen bonds with the un-ionized hydroxyl groups on cotton fabric. Therefore, the type of interaction between the cotton (cellulose) base fabric and the coatings was mainly electrostatic gravitational forces and hydrogen bonds. In the spectrum of pristine cotton fabric, the peaks at 3400 cm^−1^, 2895 cm^−1^, 1640 cm^−1^, and 1026 cm^−1^, corresponded to the O-H, C-H, O-H, and C-O in the cellulose [40]. As for the CH/APP fabric and CH/APP@TiO_2_-SiO_2_-HMDS fabric, the new absorption bands that appeared at 1533 cm^−1^ were assigned to the stretching vibration of -NH_3_^+^ belonging to the protonation of -NH_2_ in CH. Furthermore, additional absorption peaks at 1265 cm^−1^, 890 cm^−1^, and 800 cm^−1^ were clearly observed, which matched to the P=O, P-O-P in APP, Si-C in SiO_2_ and HMDS, respectively. The band at 3000–Ff water droplet on the different cotton sZ3600 cm^−1^ was attributed to the overlapping of O-H and N-H vibrations [41], whereas the form of the band grew blunt, most likely due to increasing N-H content from APP. The absorption peaks at 3480 cm^−1^, 1630 cm^−1^ were assigned to the O-H vibrations of TiO_2_, and 630 cm^−1^ or 400–700 cm^−1^ was assigned to the characteristic peak of TiO_2_ [42,43]. However, these characteristic peaks basically coincided with the relevant characteristic peaks of cellulose [44]. Therefore, it was difficult to find out the characteristic peak of TiO_2_ directly from the FTIR spectra. However, according to the EDS test results, the surface of the treated cotton fabric included Si and Ti elements, indicating that the TiO_2_-SiO_2_-HMDS composite particles were finished on the surface of the cotton fabric. Consequently, these findings suggested that CH, APP and TiO_2_-SiO_2_-HMDS were effectively applied to the surface of cotton fabric.

### 3.2. XRD

The crystal profile of the pristine cotton, CH/APP cotton, and CH/APP@TiO_2_-SiO_2_-HMDS cotton was characterized by XRD analysis. The XRD curves were shown in Figure 3b. As shown in Figure 3b, the diffraction peaks of pristine cotton at 14.7°, 16.1°, 22.4° and 34.0° correspond to the characteristic signals of cellulose [45]. The curves of CH/APP cotton, and CH/APP@TiO_2_-SiO_2_-HMDS cotton exhibited the diffraction peaks at 14.7°, 16.1°, and 22.4°, indicating the main crystal structure of fabric was not changed. However, the intensity of diffraction peaks at 14.7°, 16.1° and 22.4° decreased, and the diffraction peak at 34.0° disappeared. This could be because the surface of the cotton fabric was covered with a flame-retardant and superhydrophobic coating after finishing, which affected the diffraction peak intensity of the cotton fabric. Therefore, the diffraction peak intensity of the cotton fabric was weakened at 14.7°, 16.1° and 22.4°. However, the diffraction peak intensity of the pristine cotton was weak at 34.0° and disappeared after the coating. In addition, some new peaks appeared for the curves of the CH/APP cotton and CH/APP@TiO_2_-SiO_2_-HMDS cotton, compared with that of the pristine cotton. As shown in the patterns of CH/APP cotton, the characteristic peaks at 26.2° and 27.5°, which could be ascribed to the characteristic peaks of APP with crystalline I structure. APP had a strong diffraction peak at about 16°, which overlapped with the characteristic peaks of cellulose. The low-intensity reflections observed in CH/APP@TiO_2_-SiO_2_-HMDS cotton at 27.6°, 36.4°, 41.4°, 54.4°, and 56.7° correspond to a TiO_2_ rutile structure [46,47]. Therefore, the crystal structure of cotton was nearly unaffected and the reaction primarily occurred in the amorphous region of cotton.

### 3.3. Thermal Stability

**Figure 4:**
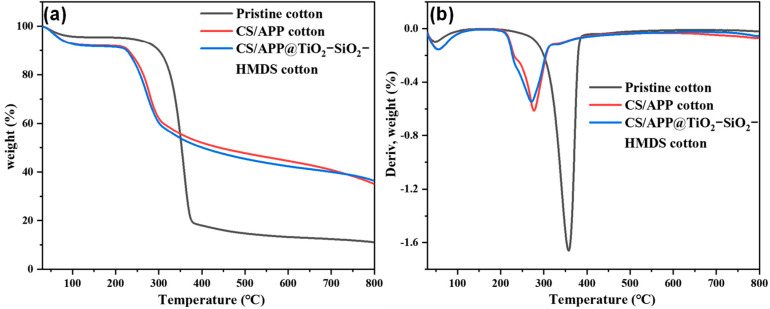
The TGA (**a**) and DTG curves (**b**) of the pristine cotton, CH/APP cotton, and CH/APP@TiO_2_-SiO_2_-HMDS cotton under nitrogen.

The TGA and DTG curves of the pristine cotton, CH/APP cotton, and CH/APP@TiO_2_-SiO_2_-HMDS cotton under nitrogen were shown in Figure 4, respectively. In addition, some characteristic parameters, such as temperature at 10% weight loss (T_10%_) and maximum weight loss (T_max_), are listed in Table 2. The char residue, T_10%_, and T_max_ of pristine cotton were 11.1%, 300 °C and 358 °C, respectively. Thermal deterioration of pristine cotton occurs mostly between 235 °C and 379 °C. Approximately 80% of the mass was lost at this step, which was attributed to the destruction of saccharide rings for cotton to produce glucose, which was then broken down into smaller molecular volatiles and residual chars [48]. The T_10%_ and T_max_ of CH/APP cotton and CH/APP@TiO_2_-SiO_2_-HMDS cotton fabric samples were much lower compared with those of the pristine cotton fabric. As for CH/APP cotton and CH/APP@TiO_2_-SiO_2_-HMDS cotton fabric, the T_10%_ decreased to 224 °C, the T_max_ decreased to 277 °C, 273 °C, and the char residue increased to 35.1 wt%, 36.42 wt%, respectively. It was mostly because APP decomposed at a lower temperature, producing polyphosphoric acid. Polyphosphoric acid could accelerate the generation of residual char by catalyzing cellulose and CH by having several hydroxyl groups. Furthermore, the increased residual char was beneficial in slowing heat/mass transmission between the matrix and its surroundings. As a result, the cellulose pyrolysis process was repressed and the char residue was enhanced at 800 °C. CH/APP cotton and CH/APP@TiO_2_-SiO_2_-HMDS cotton fabric produced fewer flammable gases and a more intumescent char layer, which helped to isolate oxygen and prevent cotton fabric further breakdown. According to TGA and DTG results, flame retardant coatings slowed down the degradation rate, minimized concentrated mass loss, and encouraged more char. [49,50,51].

### 3.4. Characterizations for Flame-Retardant Properties

**Figure 5:**
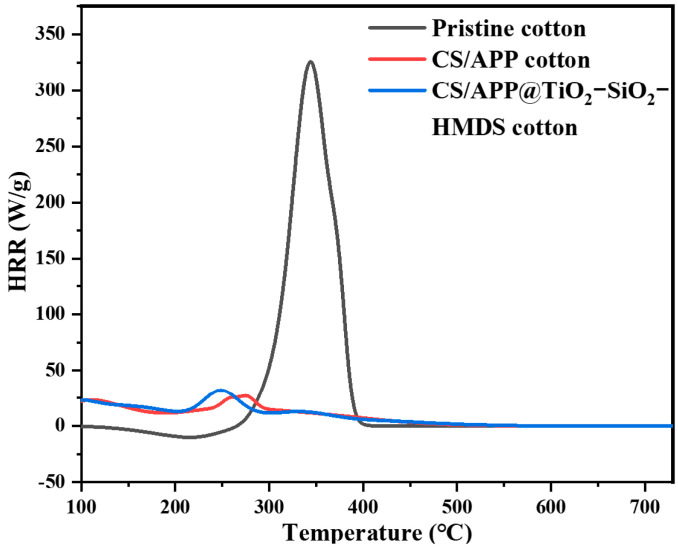
HRR curves of pristine cotton, CH/APP cotton, and CH/APP@TiO_2_-SiO_2_-HMDS cotton samples from MCC.

MCC and vertical flame tests were used to evaluate the flame-retardant properties of cotton fabric. Figure 5 depicts the typical heat release rate (HRR) curves of MCC, and the characteristic parameters are listed in Table 3. The HRR curve of the pristine cotton fabric presented a sharp peak between 300 and 400 °C, and its HR Capacity, PHRR, THR, and T_max_ values were 360 J/g-K, 327.6 W/g, 13.4 kJ/g, and 344.2 °C, respectively. The HR Capacity, PHRR, THR, and T_max_ values of CH/APP cotton were 31 J/g-K, 25.06 W/g, 5.5 kJ/g, and 257.2 °C, respectively. The HR capacity, PHRR, THR, and T_max_ values of CH/APP@TiO_2_-SiO_2_-HMDS cotton fabric were 37 J/g-K, 31.29 W/g, 5.9 kJ/g, and 241.7 °C, respectively. The values of characteristic parameters decreased significantly. This significant change indicated that the coating effectively protected the cotton fabrics by building a thermal barrier before the cotton was damaged. Meanwhile, APP in the coatings acted as the acid and gas sources by producing polyphosphoric acid, water, and ammonia. Furthermore, the synergistic effects of CH/APP enhanced the creation of more uniform and compact carbon layers on the surface of the fabric, thereby restricting the entrance of oxygen and the transmission of heat, ultimately preventing continued fabric burning [52,53,54].

**Figure 6:**
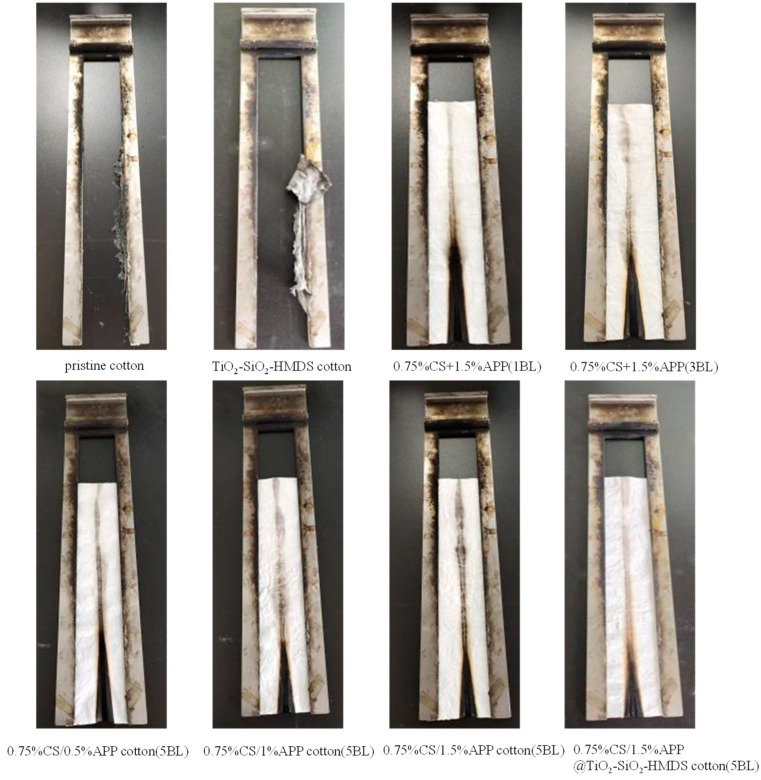
Vertical flame test optical images of cotton samples.

The combustion behavior of the uncoated and the coated cotton fabrics was assessed by vertical flame test (VFT) to describing a real fire scene. The optical images and data results of VFT were displayed in Figure 6 and Table 4. After combustion, pristine cotton fabric left only a few fragments on the holder, as seen in Figure 6. When the igniter was withdrawn from the CH/APP coated cotton fabric, the fire spread more slowly and extinguished immediately, leaving the entire sample just slightly charred. This demonstrated that the flame retardancy of cotton fabric was improved after coating. As shown in Table 4, the damaged length, burning time, afterglow of pristine cotton fabric were 30 cm, 16.25 s, 121.38 s, respectively. The damaged length, burning time, afterglow of CH/APP cotton fabric decreased to 5.7 cm, 0 s, 0 s, respectively. The damaged length, burning time, afterglow of CH/APP@TiO_2_-SiO_2_-HMDS cotton fabric decreased to 6.8 cm, 0 s, 0 s, respectively. It was remarkable that the CH/APP fabric and CH/APP@TiO_2_-SiO_2_-HMDS fabric self extinguished quickly after the fire source was removed, and they possessed good flame-retardant properties. In addition, the flame retardancy of cotton fabric changed little after TiO_2_-SiO_2_-HMDS treatment. It could be seen from Table 4 that the weight gain rate of cotton fabric increased from 7.5% to 46.0% when the number of assembly layers increased from 1 BL to 5 BL. The VFT data of 5BL CH/APP coated cotton fabric were lower than 1BL and 3BL CH/APP coated fabric. The results showed that the flame retardancy of cotton fabric was improved with the increase of weight gain rate. This was because the flame-retardant components increased on the surface of the cotton fabric when the weight gain rate increased, thereby improving the flame-retardant properties of the cotton fabric. When the amount of APP is increased from 0.5% to 1.5%, the flame retardancy of cotton fabric was improved. This was because polyphosphoric acid, which was generated from the thermal breakdown of APP, catalyzed the dehydration and carbonization of cotton fabrics, lowering the quantity of combustible gases released and creating more residual char. The remaining chars protected the matrix from heat/mass transfer, enhancing the flame retardancy of cotton textiles.

**Figure 7:**
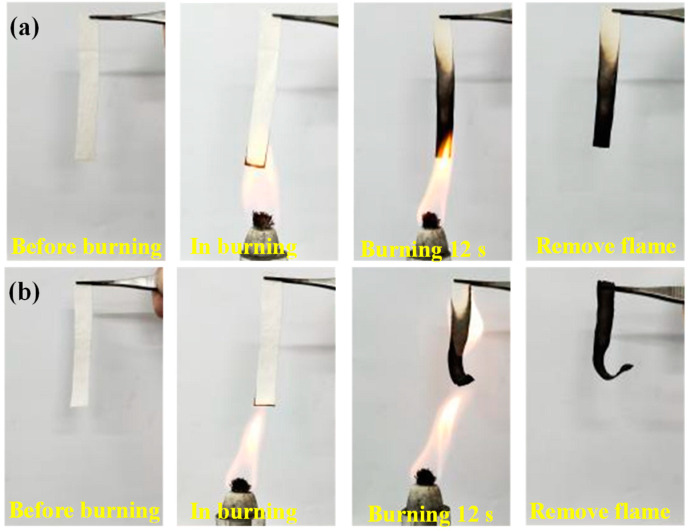
Combustion test optical images of CH/APP@TiO_2_-SiO_2_-HMDS ((**a**). 0 laundering cycle, (**b**). 50 laundering cycles).

The laundering resistance of the flame-retardant property of the fabric was investigated by direct flame ignition with an alcohol lamp, and the results are presented in Figure 7. The entire ignition process occurred in approximately 12 s. As is clear from Figure 7a, CH/APP@TiO_2_-SiO_2_-HMDS cotton immediately stopped burning after leaving the flame and only part of the cotton fabric was carbonized, indicating excellent self-extinguishing behavior. It could be seen from Figure 7b that the cotton fabric was not burned completely after leaving the flame, and all of them were carbonized. Although the carbonization length of the cotton fabric after 50 laundering cycles was greater than that of cotton fabric before laundering cycles, it could still self-extinguish after leaving the flame, indicating that it showed good flame retardancy. This was because the superhydrophobic finishing reduced the loss of flame-retardant components in the laundering cycles of cotton fabrics, and maintained the durability of flame-retardant properties. The results indicated that the flame retardancy of CH/APP@TiO_2_-SiO_2_-HMDS cotton was less affected after the 50 laundering cycles.

**Figure 8:**
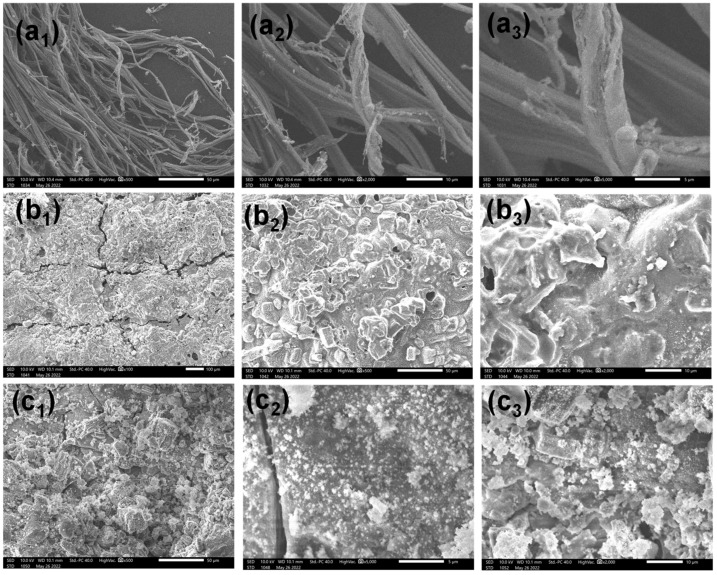
SEM images of char residues after flame exposure for 12 s of pristine cotton (**a_1_**–**a_3_**), CH/APP cotton (**b_1_**–**b_3_**), CH/APP@TiO_2_-SiO_2_-HMDS cotton (**c_1_**–**c_3_**).

The cotton fabric residues after the VFT were examined by SEM to further investigate the flame-retardant mechanism. Morphologies of pristine cotton, CH/APP cotton, and CH/APP@TiO_2_-SiO_2_-HMDS cotton are shown at various magnifications in Figure 8. After the test, the fibers of pristine cotton cracked severely and decreased in diameter, leaving some slender and incomplete char remnants, as illustrated in Figure 8(a_1_–a_3_). As for CH/APP and CH/APP@TiO_2_-SiO_2_-HMDS coated cotton fabric, the char surface was very thick, dense and continuous, and some parts of the surface were intumescent (Figure 8(b_1_–b_3_,c_1_–c_3_)). Meanwhile, the char residues remained solid and almost unbroken, with just a few minor fractures of partial fibers and almost no shrinkage in diameter. Furthermore, some tiny particles were found on the intumescent char layer of the CH/APP@TiO_2_-SiO_2_-HMDS cotton fabric surface, which was rougher than that of the CH/APP coated cotton fabric. It might be due to the combustion of TiO_2_-SiO_2_-HMDS. These phenomena were caused by the intumescent action of CH and cellulose with a high concentration of hydroxyl groups as the char agent and APP as the acid and gas agent. When cotton fibers coated with APP and CH caught fire, they created intumescent chars that prevented the matrix from burning further, improving the flame retardancy of the coated cotton fabric.

### 3.5. Superhydrophobicity Measurement

**Figure 9:**
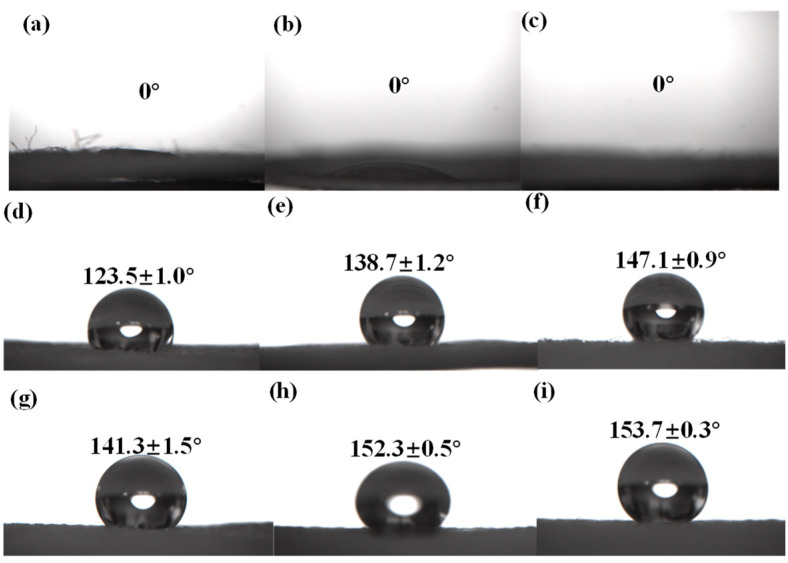
Photographic images of water droplet on the different cotton samples surface. Pristine cotton (**a**), CS/APP cotton (**b**), 0.15 g TiO_2_ amounts of TiO_2_-SiO_2_-HMDS cotton (**c**), different TiO_2_ amounts ((**d**). 0.15 g, (**e**). 0.45 g, (**f**). 0.75 g) of CH/APP@TiO_2_-SiO_2_-HMDS cotton, different HMDS amounts ((**g**). 3 mL, (**h**). 6 mL, (**i**). 7.5 mL) of CH/APP@TiO_2_-SiO_2_-HMDS cotton.

Photographic images of a water droplet on the different cotton samples surface are presented in Figure 9. In Figure 9, the water droplet was fully spread on the surface of the pristine cotton fabric and the CA was 0°. The water droplet was also fully spread on the surface of the CH/APP coated cotton and TiO_2_-SiO_2_-HMDS cotton fabric, and the CA was 0°. However, the water droplet was spherical on the surface of the CH/APP@TiO_2_-SiO_2_-HMDS cotton fabric, and the CA was 123.5°. It indicated that cotton fabrics treated by CH/APP or TiO_2_-SiO_2_-HMDS alone have no hydrophobic properties. In addition, the CH/APP@TiO_2_-SiO_2_-HMDS cotton fabric had good hydrophobicity. Figure 9d–i showed the effect of TiO_2_ and HMDS doses on the CA of SFR cotton fabric. It could be seen that the CA grew progressively with the increase of TiO_2_ and HMDS dosage. When the dosage of TiO_2_ and HMDS was 0.15 g and 3ml, the CA were 123.5° and 141.3°, respectively. When the dosage of TiO_2_ and HMDS increased to 0.75 g and 7.5 mL, the CA of cotton fabric reached 147.1° and 153.7°, respectively. It indicated that the hydrophobic properties of cotton fabric were improved with the increase dosage of TiO_2_ and HMDS. The CA measurement result showed that the CH/APP@TiO_2_-SiO_2_-HMDS cotton fabric had excellent superhydrophobic properties.

**Figure 10:**
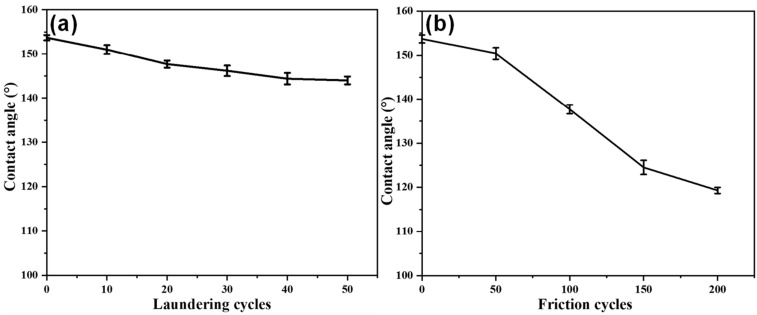
Effect of laundering cycles (**a**) and friction cycles (**b**) times on the CA of the CS/APP@TiO_2_-SiO_2_-HMDS.

Durability against repeated laundering and abrasion resistance were the important requirements for multifunctional fabric. Therefore, the hydrophobic property of laundering and abrasion resistance of the fabric were evaluated. Figure 10 shows that the CS/APP@TiO_2_-SiO_2_-HMDS cotton fabric could keep superhydrophobicity after 10 laundering cycles and 50 friction cycles. In Figure 10a, the superhydrophobic surface was turned into a hydrophobic surface after 10 laundering cycles. This was due to the decrease of micro/nano particles on the surface of the cotton fabric after repeated laundering, which led to the decrease of hydrophobicity on the cotton fabric. However, the CA of the cotton fabric was kept at about 144° after 50 laundering cycles, indicating that the CS/APP@TiO_2_-SiO_2_-HMDS cotton fabric could withstand certain laundering, and still maintained good hydrophobicity. As seen from Figure 10b, after 50 friction cycles the superhydrophobic surface was turned into hydrophobic surface, and the CA was decreased greatly. This was because the micro/nano particles and fiber on the surface of the cotton fabric were partially damaged after multiple friction cycles. The CA of the CS/APP@TiO_2_-SiO_2_-HMDS cotton fabric was about 119° after 200 friction cycles, showing that the fabric was still hydrophobic. The laundering and abrasion resistance results showed that the durability of superhydrophobicity of CS/APP@TiO_2_-SiO_2_-HMDS cotton fabric was good.

### 3.6. Wettability, Self-Cleaning Property and UV-Shielding Property

**Figure 11:**
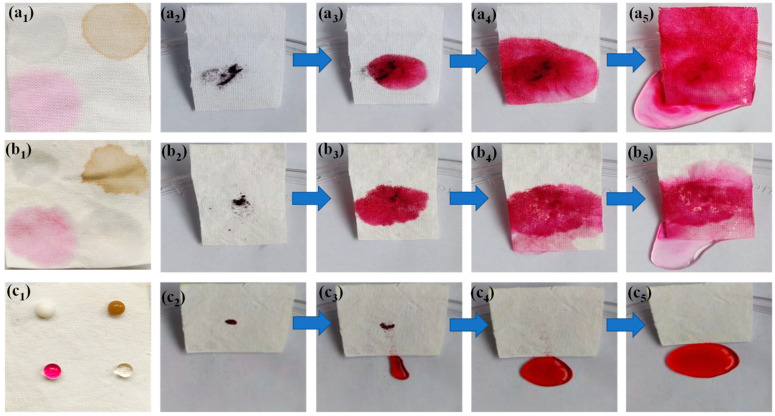
Photographs of antifouling ability test and self-cleaning property test for pristine cotton fabric (**a_1_–a_5_**), CS/APP cotton fabric (**b_1_–b_5_**) and CS/APP@TiO_2_-SiO_2_-HMDS cotton fabrics (**c_1_–c_5_**).

Wettability and self-cleaning properties of SFR cotton fabric were important in practical applications. Figure 11(a_1_–c_1_) shows the surface wettability of cotton materials. As shown in Figure 11(a_1_), pristine cotton fabric was inherently hydrophilic, and the droplets easily spread on the surface. The droplets spread quickly on the CH/APP coated cotton fabric surface, as seen in Figure 11(b_1_). On the other hand, droplets could keep a stable spherical form on the surface of the CH/APP@TiO_2_-SiO_2_-HMDS textiles.

As shown in Figure 11(a_2_–a_5_), when the fabric containing reactive red dye powders was tilted at an angle of about 20° and washed with water droplets, the water quickly infiltrated the fabric while the reactive red dye powders remained on the surface. The same test phenomenon on the CH/APP coated cotton fabric was observed as on pristine cotton fabric (Figure 11(b_2_–b_5_)). On the other hand, the water droplets simply rolled down the SFR cotton fabric while removing dirt without any further external action, keeping the cloth clean and dry (Figure 11(c_2_–c_5_)). It indicated that pristine cotton and CH/APP coated cotton fabrics had no antifouling and self-cleaning properties. The results showed that CH/APP@TiO_2_-SiO_2_-HMDS cotton fabric had the outstanding antifouling and self-cleaning properties.

Textiles with UV-shielding properties can provide a significant barrier for human skin, preventing skin damage and aging. The UV-shielding property of cotton was studied by the UPF value, and both UVA and UVB transmittance, and the results are shown in Table 5. As displayed in Table 5, the CH/APP cotton had a much higher UPF value and remarkably lower T-UVA and T-UVB transmittance in comparison with pristine cotton. This was because the CH and APP were deposited on the surface of the cotton fabric through the LBL assembly method, and a coating was formed on the surface of the cotton fabric, which increased the thickness of the cotton fabric and reduced the gap between the cotton fibers, thereby reducing the transmittance of ultraviolet light. The UV-shielding property of CH/APP@TiO_2_-SiO_2_-HMDS cotton was better than that of the CH/APP cotton fabric, indicating the important role that TiO_2_-SiO_2_-HMDS played in improving the UV-shielding property of the cotton. This was because the TiO_2_ had a high refractive index and UV-ray absorption rate, imparting excellent UV shielding to the cotton fabrics. Therefore, the result indicated that CH/APP@TiO_2_-SiO_2_-HMDS cotton had excellent UV-shielding properties.

Furthermore, the preparation and performance of the various superhydrophobic, flame-retardant cotton fabrics were compared with the previous works, as shown in Table 6. LBL technique is a common method for producing flame-retardant cotton fabric. LBL assembly was a simple and versatile technique that could be applied to various polymers, colloids or molecules for the formation of a thin film, which could equip the substrates with different functionalities. However, the flame-retardant components could be lost during washing or friction. In addition, the preparation of superhydrophobic cotton fabric was achieved by constructing the rough structure of TiO_2_. Some reports disclosed that nano TiO_2_ could catalyze the depolymerization of fabric substrates and shorten their lifespan, severely restricting practical applications of the functionalized fabric composites. As can be seen from Table 6, the preparation process of superhydrophobic flame retardant textiles was complicated. In addition, the durability of superhydrophobic and flame-retardant textiles prepared by the one-pot method needed to be improved. In this work, the flame retardancy of cotton fabric was endowed by the LBL method. The hydrophobic TiO_2_-SiO_2_-HMDS particles were prepared by the sol–gel method, and then the superhydrophobic finishing of cotton fabric was carried out by the one-step method, which saved steps in the process. It was helpful to maintain the flame retardancy for a long term. TiO_2_-SiO_2_-HMDS not only endowed fabrics with excellent UV-shielding properties, but also reduced the photocatalytic performance of TiO_2_ and protected the low-surface-energy materials and fabrics. It was observed that the TiO_2_-SiO_2_-HMDS cotton fabrics maintained surface superhydrophobicity after undergoing 10 laundering cycles and 50 abrasion cycles.

## 4. Conclusions

In conclusion, a superhydrophobic and flame-retardant coating was developed onto cotton fabric with CH, APP and TiO_2_-SiO_2_-HMDS composite via facile layer-by-layer assembly and dip-coating. SEM, EDS, and FTIR were used to characterize the fabric, which proved that there are strong chemical bonds in the coating. According to TGA, VFT, and MCC data, the deposition of CH and APP improves thermal stability, flame retardancy, and combustion behaviors. The SFR cotton fabric not only exhibited superhydrophobicity with a contact angle of 153.7°, but also possessed excellent self-cleaning, antifouling and UV-shielding properties. More importantly, the superhydrophobic properties of cotton fabrics are resistant to 10 laundering cycles or 50 friction cycles. The fabrication of such an SFR coating on cotton fabric is simple, low-cost, and efficient, indicating considerable potential for large-scale manufacturing and deployment of multifunctional superhydrophobic and flame-retardant materials.

## Figures and Tables

**Table 1:** The elemental content in weight % of pristine cotton and treated cotton.

Samples	C	O	N	P	Si	Ti
	Weight %	Weight %	Weight %	Weight %	Weight %	Weight %
pristine cotton	35.32	64.68	0	0	0	0
CH/APP cotton	7.57	56.15	2.06	34.23	0	0
CH/APP@TiO_2_-SiO_2_-HMDS cotton	0.14	43.58	0	0	27.90	28.38

**Table 2:** TGA and DTG curves characteristic parameters for cotton fabrics.

Samples	T_10%_ (°C)	T_max_ (°C)	Residue at 800 °C (wt %)
pristine cotton	300	358	11.1
CH/APP cotton	224	277	35.1
CH/APP@TiO_2_-SiO_2_-HMDS cotton	224	273	36.4

**Table 3:** Microscale combustion calorimeter results for various samples.

Samples	HR Capacity/(J/g-K)	PHRR/(W/g)	THR/(kJ/g)	T_max_(°C)
pristine cotton	360	327.6	13.4	344.2
CH/APP cotton	31	25.06	5.5	257.2
CH/APP@TiO_2_-SiO_2_-HMDS cotton	37	31.29	5.9	241.7

**Table 4:** Vertical flame test data of the uncoated and coated fabrics.

Samples	Weight Gain Rate (%)	Damaged Length (Horizontal) (cm)	Burning Time (s)	Afterglow (s)
pristine cotton	-	30	16.25	121.38
TiO_2_-SiO_2_-HMDS cotton	-	30	19.47	24.84
0.75%CH + 1.5%APP (1BL)	7.5%	10.3	0	0
0.75%CH + 1.5%APP (3BL)	30.5%	7.8	0	0
0.75%CH/0.5%APP cotton (5BL)	-	7.8	0	0
0.75%CH/1%APP cotton (5BL)	37.0%	6.5	0	0
0.75%CH/1.5%APP cotton (5BL)	46.0%	5.7	0	0
0.75%CH/1.5%APP@TiO_2_-SiO_2_-HMDS cotton (5BL)	-	6.8	0	0

**Table 5:** UV-shielding properties of cotton fabric samples.

Samples	UPF	Transmittance (%)	
		UVA	UVB
pristine cotton	15.53	6.39	6.24
CH/APP cotton	165.69	0.68	0.52
CH/APP@TiO_2_-SiO_2_-HMDS cotton	825.81	0.14	0.11

**Table 6:** Comparison of preparation and performance of various superhydrophobic, flame-retardant cotton fabrics reported by other researchers and in this work *.

Fabrics	Method	Properties	Durability		Refs.
			Laundering	Friction	
APP/AM/CS coated cotton fabric	LBL	flame-retardant	-	-	[49]
SiO_2_-PEI/PA coated cotton fabric	LBL	flame-retardant	-	-	[55]
PTES- TiO_2_ coated cotton fabric	one-pot hydrothermal reaction	Superhydrophobic UV-shielding	5	<30	[56]
TiO_2_ coated cotton fabric	sol-gel process	flame-retardant	-	>2100	[57]
C3-PDMS-TiO_2_ cotton fabrics	two-step spraying method	flame-retardant, superhydrophobic	5	10	[58]
Ag/Cu–DMTD- ODTS coated cotton fabric	Trilayer coatings immersion technique.	flame-retardant superhydrophobic	<10	1	[59]
APP-PDMS-silica coated cotton fabric	one-pot approach via sol-gel reaction	Superhydrophobic flame-retardant	-	-	[27]
CS/APP@TiO_2_-SiO_2_-HMDS coated cotton fabric	LBL and sol-gel process	Superhydrophobic flame-retardant UV-shielding	10	50	This work

* -Not reported.

## Data Availability

The data presented in this study are available on request from the corresponding author.
